# Sparse regressions for predicting and interpreting subcellular localization of multi-label proteins

**DOI:** 10.1186/s12859-016-0940-x

**Published:** 2016-02-24

**Authors:** Shibiao Wan, Man-Wai Mak, Sun-Yuan Kung

**Affiliations:** Department of Electronic and Information Engineering, The Hong Kong Polytechnic University, Hong Kong, SAR China; Department of Electrical Engineering, Princeton University, New Jersey, USA

## Abstract

**Background:**

Predicting protein subcellular localization is indispensable for inferring protein functions. Recent studies have been focusing on predicting not only single-location proteins, but also multi-location proteins. Almost all of the high performing predictors proposed recently use gene ontology (GO) terms to construct feature vectors for classification. Despite their high performance, their prediction decisions are difficult to interpret because of the large number of GO terms involved.

**Results:**

This paper proposes using sparse regressions to exploit GO information for both predicting and interpreting subcellular localization of single- and multi-location proteins. Specifically, we compared two multi-label sparse regression algorithms, namely multi-label LASSO (mLASSO) and multi-label elastic net (mEN), for large-scale predictions of protein subcellular localization. Both algorithms can yield sparse and interpretable solutions. By using the one-vs-rest strategy, mLASSO and mEN identified 87 and 429 out of more than 8,000 GO terms, respectively, which play essential roles in determining subcellular localization. More interestingly, many of the GO terms selected by mEN are from the biological process and molecular function categories, suggesting that the GO terms of these categories also play vital roles in the prediction. With these essential GO terms, not only where a protein locates can be decided, but also why it resides there can be revealed.

**Conclusions:**

Experimental results show that the output of both mEN and mLASSO are interpretable and they perform significantly better than existing state-of-the-art predictors. Moreover, mEN selects more features and performs better than mLASSO on a stringent human benchmark dataset. For readers’ convenience, an online server called SpaPredictor for both mLASSO and mEN is available at http://bioinfo.eie.polyu.edu.hk/SpaPredictorServer/.

## Background

Within living organisms, proteins need to locate in the right subcellular compartments to perform various biological functions. Mislocalized human proteins are liable to cause numerous human diseases, such as kidney stone [1], Bartter syndrome [2], primary human liver tumors [3], Alzheimer’s disease [4], breast cancer [5], pre-eclampsia [6] and minor salivary gland tumors [7]. Knowing where a protein resides within a cell is an indispensable and essential step to uncover its functions and detect drug targets [8]. Traditional wet-lab techniques such as cell fractionation, electron microscopy and fluorescent microscopy imaging, are applied to construct high quality localization databases such as the Human Protein Atlas.^1^ However, the processes are laborious, expensive and time-consuming. To tackle tremendous numbers of newly discovered protein sequences generated by large-scale sequencing projects, efficient computational methods are required for fast and accurate prediction of protein subcellular localization (PSCL).

Conventionally, PSCL was tackled by sequence-based approaches. This type of approaches includes three categories: (1) sorting-signals based methods [9–11]; (2) amino-acid composition-based methods [12–15]; (3) homology-based methods [16, 17]. Beyond sequence information, knowledge-based approaches have been developed. This type of approaches uses information from knowledge databases, such as Gene Ontology (GO)^2^ terms [18–28], PubMed abstracts [29, 30], or Swiss-Prot keywords [31, 32]. Among these methods, GO-based methods were found to be superior in terms of performance [23, 33–35].

Because many studies [36–39] have found the prevalence of multi-location proteins in living organisms, recent studies have been focusing on predicting not only single-location proteins, but also multi-location proteins. Multi-label proteins are found to participate in various metabolic activities in multiple cellular compartments. For example, the glucose transporter GLUT4 is found in both the plasma membrane and the intracellular vesicles of adipocytes [40, 41]; proteins involved in fatty acid *β*-oxidation is found in the mitochondria and peroxisome; and antioxidant defense proteins are known to reside in the peroxisome, cytosol and mitochondria [42].

Many state-of-the-art multi-label predictors – such as iLoc-Hum [27], Hum-mPLoc 2.0 [43], mGOASVM [44], HybridGO-Loc [45], R3P-Loc [46], mPLR-Loc [47] and others [48–50] – use GO information as features and apply different multi-label classifiers to tackle the multi-label classification problem. Nevertheless, due to the high dimensionality of GO features, these GO-based predictors often have the following drawbacks:

1. **Lack of interpretability**. These predictors can only give insights into where the query proteins are located, but cannot provide biological reasons of why they reside there. This is possibly a common problem for most machine-learning based approaches, because it is usually difficult to correlate the statistical characteristics of biological data with biological phenomena. On the other hand, biologists want to know not only the prediction results of query proteins, but also biological features or factors that lead to the prediction results. Therefore, the lack of interpretability may limit the powers and applications of these predictors. As far as we know, there is only one subcellular-location predictor called YLoc [51] that is interpretable. However, YLoc requires heterogeneous biological features such as sorting signals, PROSITE^3^ patterns and GO terms, which are not always available for every protein.
2. **Susceptibility to overfitting**. The number of extracted GO features from knowledge databases (e.g., GO annotation database^4^) is considerably larger than the number of proteins of interest. Most of the existing predictors (except R3P-Loc) construct feature vectors with dimensions as high as several thousand. Among these thousands of features, it is likely that many are irrelevant or redundant, causing the predictors suffer from overfitting

To tackle the problems mentioned above, this paper proposes two sparse and interpretable multi-label predictors, namely **mLASSO** and **mEN** for large-scale predictions of both single- and multi-location proteins. Given a query protein sequence, a set of GO terms are retrieved from two newly created compact databases by the procedures described in [46]. The frequencies of GO occurrences are used to formulate frequency vectors with dimension over 8000. By using a one-vs-rest LASSO-based (least absolute shrinkage and selection operator-based) classifier and an EN-based (elastic net) classifier, 87 and 429 out of these 8,000+ GO terms were selected, respectively. With the selected GO terms, the frequency vectors are converted into dimension-reduced feature vectors (87-dim for mLASSO and 429-dim for mEN). Subsequently, the 87-dim (429-dim resp.) feature vectors are classified by a multi-label LASSO (EN resp.) classifier. Experimental results based on a stringent human benchmark dataset demonstrate that the two proposed predictors substantially outperform other state-of-the-art predictors. More significantly, based on GO terms selected by either mLASSO or mEN, researchers can decide not only where a protein resides within a cell, but also why it is located there. Moreover, mEN not only selects more GO terms than mLASSO, but also performs better than mLASSO. We have also found that besides cellular-component GO terms, GO terms from the categories of molecular functions and biological processes also contribute to the final predictions.

## Legitimacy of using GO information

Some researchers may have reservations about the use of GO information for PSCL. In the following, we list the concerns (C1 and C2) and our explanations (E1 and E2) of why these concerns do not cause problems in PSCL.

1. Because the cellular component GO terms have already been annotated with cellular component categories, GO-based methods can be simply replaced by a lookup table using the cellular component GO terms as the keys and the component categories as the hashed value.
2. This naive solution is not recommended because not only the cellular component GO terms are relevant to the PSCL, GO terms from the biological processes and molecular functions also play important roles, as demonstrated in [52]. In particular, it has been found [52] that GO terms in biological process and molecular function categories are particularly relevant to nucleus, extracellular space, membrane, mitochondrion, endoplasmic reticulum and Golgi apparatus. In fact, the relationship between GO terms and PSCL is not a one-to-one mapping and recent studies [34, 44] have already shown that this naive solution will lead to very poor performance.
3. Are GO-based methods equivalent to transferring annotations from BLAST [53] homologs?
4. This concern is explicitly addressed in our previous study [34], which demonstrated that GO-based methods remarkably outperform methods that only use BLAST and homologous transfer (in Table 4 of [34]). Besides, Briesemeister et al. [54] also found that using BLAST alone is not sufficient for reliable prediction.

In fact, as suggested by Chou [55], given a predictor, as long as its inputs are amino acid sequences and its outputs (predictions) are subcellular localizations, the predictions made by the predictor are legitimate; whether the predictor uses GO-based methods or non GO-based methods is not an issue. Some other papers [56, 57] also provide strong evidences supporting the legitimacy of using GO information for subcellular localization. In particular, as suggested by [57], the good performance of GO-based methods is due to the fact that the feature vectors in the GO space can better reflect their subcellular locations than those in the Euclidean space or any other simple geometric space.

## Results

### Datasets

**Fig. 1:**
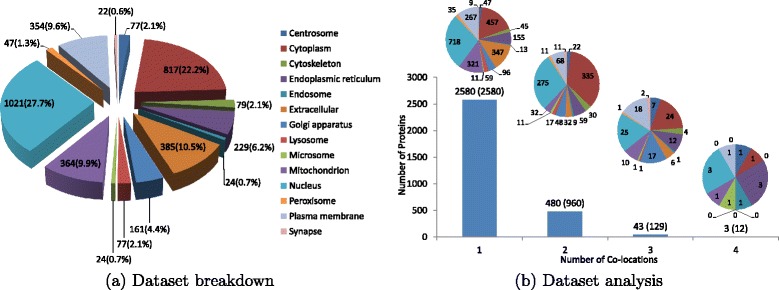
Information of the human dataset. (**a**) Dataset breakdown; (**b**) dataset analysis. The number of proteins shown in each subcellular location represents the number of ‘locative proteins’ [44, 65]. The dataset comprises 3106 actual proteins and 3681 locative proteins distributed in 14 subcellular locations. In (**b**), for each bar, the numbers *m*(*n*) on top denote that there are *m* actual proteins and *n* locative proteins having the same number of co-location(s) indicated in the bottom of the bar. For example, there are 43 actual and 129 locative proteins that have three subcellular locations. The small pie charts show the distribution of locative proteins having the number of co-location(s) shown in the bottom of the bar chart

A stringent human benchmark dataset [43] was used to evaluate the performance of mLASSO and mEN. The human dataset was created from Swiss-Prot 55.3, which is a publicly accessible protein database.^5^ This benchmark dataset is downloadable from the hyperlink in the SpaPredictor server. It contains 3106 human proteins distributed in 14 locations. The sequence identity of the dataset was cut off at 25 %. Figure 1(a) shows the breakdown of the human dataset. Here, 3106 actual proteins [44] correspond to 3681 locative proteins [44, 58].^6^ As can be seen from Fig. 1(a), the majority (79.9 %) of the human proteins are located in cytoplasm, nucleus, extracellular, mitochondrion and plasma membrane, while proteins located in the other 9 subcellular locations account for around 20 %. This means that the dataset is very imbalanced.

Figure 1(b) further shows the distribution of co-located proteins. As can be seen, of the 3106 proteins, 2580 belong to one subcellular location, 480 belong to two locations, 43 belong to three locations, 3 belong to four locations and none to five or more locations. As shown in the pie charts in Fig. 1(b), the majority of single-location proteins are located in cytoplasm, extracellular, mitochondrion, nucleus and plasma membrane, which is consistent with the distribution of the overall locative proteins shown in Fig. 1(a). For the proteins locating at two subcellular locations, around two thirds of proteins are distributed in cytoplasm and nucleus; on the contrary, proteins residing in three or four subcellular locations are more evenly distributed than those single-location and two-location proteins. This analysis suggests that single-location proteins play more significant roles in shaping the overall distribution of the dataset; however, multi-location proteins also constitute a considerable percentage of the dataset.

### Performance metrics

To facilitate performance comparisons in multi-label classification, some sophisticated performance metrics are introduced here to better reflect the multi-label capabilities of classifiers. These measures include *Accuracy, Precision, Recall, F1-score (F1)* and *Hamming Loss (HL)*. The definitions of these five measurements for multi-label classification can be found in [45, 46]. *Accuracy, Precision, Recall* and *F1* indicate the classification performance. The higher the measures, the better the prediction performance. Among them, *Accuracy* is the most commonly used criteria. *F1-score* is the harmonic mean of *Precision* and *Recall*, which allows us to compare the performance of classification systems by taking the trade-off between *Precision* and *Recall* into account. The *Hamming Loss (HL)* [59, 60] is different from other metrics in that the former concerns about the misclassified instance-label pairs whereas the latter are more interested in the correctly classified instance-label pairs. The lower the *HL*, the better the prediction performance. More details about the performance metrics can be found in Section S7 of supplementary materials.

Since partial matching has been widely used in measuring classification performance [61, 62], especially in multi-label classification [63], we have also used another two measures: *Micro F-measure (Micro F1)* and *Macro F-measure (Macro F1)*. The definitions of these two measures in multi-label learning scenarios can be found in [63]. To compute *Macro F1*, the F1 of individual classes are independently computed and then averaged. As a result, *Macro F1* treats the F1 of individual classes equally important and the measure is insensitive to the imbalance in class sizes. On the other hand, to compute *Micro F1*, the true-positive, true-negative, and false-negative are accumulated across all classes, followed by plugging these values into the standard formula for computing F1. Therefore, *Macro F1* considers every binary decision equally important, whereas *Micro F1* will be heavily dependent on the decisions (both correct and incorrect) on the large classes [64].

Two additional measures [44, 65] are often used in multi-label subcellular localization prediction. They are overall locative accuracy (*OLA*) and overall actual accuracy (*OAA*). Specifically, denote  and  as the true label set and the predicted label set for the *i*-th protein , respectively.^7^ Then, *OLA* is given by: 
(1)

and the overall actual accuracy (*OAA*) is: 
(2)

where 
(3)

Among all the metrics mentioned above, *OAA* is the most stringent and objective. This is because if some (but not all) of the subcellular locations of a query protein are correctly predicted, the numerators of the other five measures (including *Accuracy, Precision, Recall, F1* and *OLA*) are non-zero, whereas the numerator of *OAA* in Eq. 2 is 0 (thus making no contribution to the frequency count).

Leave-one-out cross validation (LOOCV) is considered to be the most rigorous and bias-free procedure [66] for evaluating classifiers’ performance. Hence, LOOCV was used to examine the performance of mLASSO and mEN.

### Statistical analysis of the essential GO terms

**Fig. 2:**
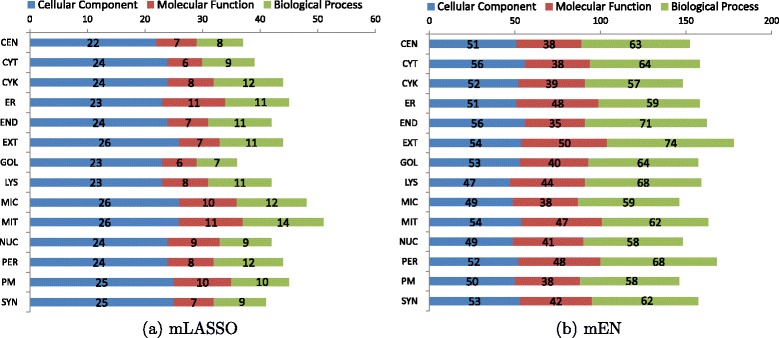
The categorical breakdown of the essential GO terms in each subcellular location for (**a**) mLASSO and (**b**) mEN. *CEN*: centrosome; *CYT*: cytoplasm; *CYK*: cytoskeleton; *ER*: endoplasmic reticulum; *END*: endosome; *EXT*: extracellular; *GOL*: Golgi apparatus; *LYS*: lysosome; *MIC*: microsome; *MIT*: mitochondrion; *NUC*: nucleus; *PER*: peroxisome; *PM*: plasma membrane; *SYN*: synapse

Figure 2(a) and (b) show the categorical breakdown of essential GO terms found by mLASSO and mEN. Figure 2(a) shows that for each subcellular location, around 30 ∼50 essential GO terms determine where a protein resides. For example, for *cytoplasm*, 39 essential GO terms contribute to the final decisions, of which 24 belong to the cellular-component category; the remaining 6 and 9 belong to molecular function and biological process categories, respectively. Besides, around half of the essential GO terms belong to cellular components, e.g., 22 out of 37 in centrosome, 24 out of 39 in cytoplasm, etc. The results indicate that for mLASSO, cellular component GO terms contribute more to the final prediction than the other GO terms. However, as shown in Fig. 2(b), the percentage of cellular-component GO terms found by mEN is much smaller. For example, for *cytoplasm*, only around 36 % (56 out of 158) belongs to cellular components. These results suggest that essential GO terms from biological processes and molecular functions may contribute more to the final predictions for mEN than for mLASSO.

A comparison between Fig. 2(a) and (b) reveals that the number of essential GO terms selected by mEN is much larger than that selected by mLASSO for all of the 14 subcellular locations; this phenomenon also occurs across the three GO categories. This is because GO terms from the same category are not independent on each other; instead they are related in a hierarchical manner. Compared to mLASSO, mEN encourages selecting correlated features together, thus causing more essential GO terms to be selected. The results are consistent with the claims by Zou and Hastie [67].

**Fig. 3:**
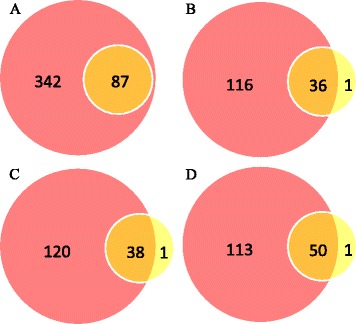
Overlapping between the essential GO terms found by mLASSO (*yellow*) and mEN (*pink*). (**a**) All subcellular locations, (**b**) centrosome, (**c**) cytoplasm and (**d**) mitochondrion

We used the degree of overlapping among the essential GO terms found by mLASSO and mEN to investigate the relationship between the GO terms found by these algorithms. The results are shown in Fig. 3. Figure 3(a) shows that the 87 essential GO terms selected by mLASSO are totally included in the 429 essential GO terms selected by mEN. Figure 3(b–d) show that for *centrosome, cytoplasm* and *mitochondrion*, there are 36, 38 and 50 overlapped essential GO terms. For each of these subcellular locations, there is only one essential GO term that is found by mLASSO but missed by mEN. Specifically, for *centrosome*, the GO term missed by mEN is GO:0005829 (CC, cytosol); for *cytoplasm*, the missed GO term is GO:0005524 (MF, ATP binding); and for *mitochondrion*, the missed GO term is GO:0005654 (CC, nucleoplasm). As can be seen from Section S1 of supplementary materials, the weights of these three GO terms for mLASSO in the aforementioned subcellular locations are negative and inconsiderable. Specifically, the weight of GO:0005829 for *centrosome* is −0.0045; the weight of GO:0005524 for *cytoplasm* is −0.0009; and the weight of GO:0005654 for *mitochondrion* is −0.0059. Therefore, even though mLASSO selects them, these GO terms play insignificant roles in predictions. We notice that GO:0005829 (CC, cytosol) is the part of cytoplasm that does not contain organelles, which, in other words, has no direct correlation with *centrosome*. Because mEN tends to select correlated features together, it is reasonable that mEN does not select GO:0005829 for *centrosome*. Similar reasons are also applied to the other two cases. The remaining 11 subcellular locations have similar situations as Fig. 3(a), meaning that all of the essential GO terms selected by mLASSO are also selected by mEN. The results suggest that mEN can select almost all the information selected by mLASSO, and more importantly, mEN can incorporate extra feature information missed by mLASSO.

### Significance of location-specific GO terms

**Fig. 4:**
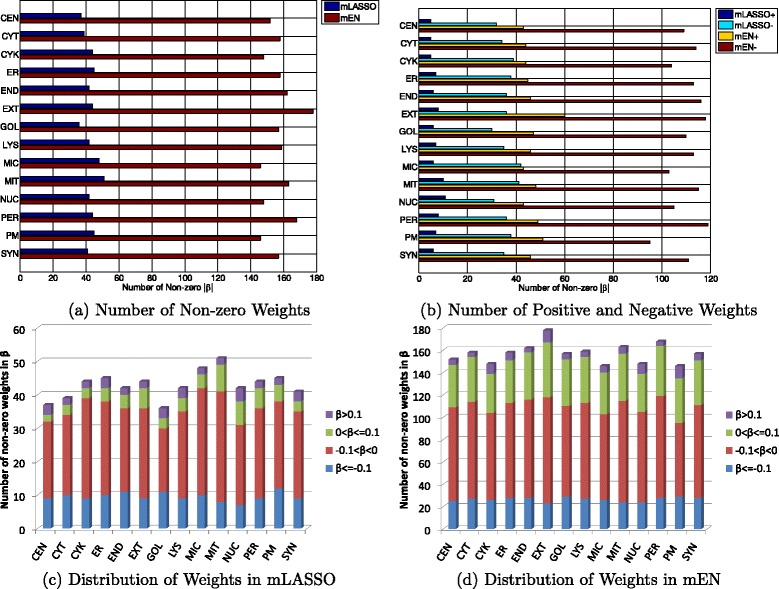
Analysis of location-specific weights *β*_*s*,*m*_ of the essential GO terms for mLASSO and mEN. (**a**) Number of non-zero weights for mLASSO and mEN; (**b**) number of positive and negative weights; (**c**) distribution of non-zero weights for mLASSO; (**d**) distribution of non-zero weights for mEN. *CEN*: centrosome; *CYT*: cytoplasm; *CYK*: cytoskeleton; *ER*: endoplasmic reticulum; *END*: endosome; *EXT*: extracellular; *GOL*: Golgi apparatus; *LYS*: lysosome; *MIC*: microsome; *MIT*: mitochondrion; *NUC*: nucleus; *PER*: peroxisome; *PM*: plasma membrane; *SYN*: synapse

To quantitatively demonstrate how and to what extent essential GO terms contribute to the prediction of subcellular locations, we analyzed the location-specific weights $\{\tilde {\boldsymbol {\beta }}_{m}\}_{m=\{1,\ldots,M\}}$ defined in Eqs. 12 and 13 for the essential GO terms.^8^ As shown in Fig. 4, the analyses include (a) the number of non-zero weights for both algorithms, (b) the number of positive and negative weights for both algorithms, (c) distribution of weights for mLASSO and (d) distribution of weights for mEN. For simplicity, *β*_*s*,*m*_ is abbreviated as *β* in the figures.

As can be seen from Fig. 4(a), for every subcellular location, the number of non-zero weights for mEN is larger than that for mLASSO, which is consistent with the results in the last section. In Fig. 4(b), we can see that for both mLASSO and mEN, the number of positive weights in every subcellular location is much smaller than that of negative weights. This result suggests that the majority of essential GO terms are indicative of not residing in a particular subcellular location.

To further analyze the significance of the non-zero weights of essential GO terms, the weights are divided into four intervals:

(4) $$ \left\{ \begin{array}{llll} \beta \leq -0.1, \\ -0.1 < \beta < 0, \\ 0 < \beta \leq 0.1, \\ \beta > 0.1. \end{array} \right.  $$

The distributions of the non-zero weights for mLASSO and mEN are shown in Fig. 4(c) and (d), respectively. From these figures, we can observe that the number of weights in the interval *β*>0.1 takes up the smallest percentage and most of the weights are within the interval of −0.1<*β*<0. This means that the number of essential GO terms indicating the presence of query proteins in a particular location is small. Nonetheless, when comparing Fig. 4(c) and (d), we found that the percentage of weights in the interval of 0<*β*≤0.1 for mEN is larger than that for mLASSO, which suggests that there are more positive indicators for mEN than for mLASSO to indicate that a query protein locates in a particular subcellular location.

**Fig. 5:**
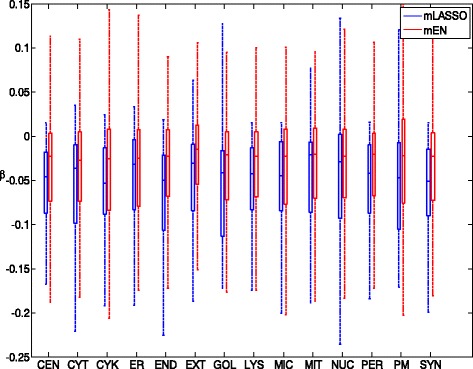
The distribution of non-zero weights in each subcellular location for mLASSO and mEN. *CEN*: centrosome; *CYT*: cytoplasm; *CYK*: cytoskeleton; *ER*: endoplasmic reticulum; *END*: endosome; *EXT*: extracellular; *GOL*: Golgi apparatus; *LYS*: lysosome; *MIC*: microsome; *MIT*: mitochondrion; *NUC*: nucleus; *PER*: peroxisome; *PM*: plasma membrane; *SYN*: synapse

To further compare mEN and mLASSO, Fig. 5 shows the range of the non-zero weights for each subcellular location. Evidently, for almost all subcellular locations, the mean weights of mEN are larger than those of mLASSO, which suggests that mEN are more capable of positively predicting a query protein than mLASSO.

We observe that among the essential GO terms, some have much larger absolute weights (i.e. |*β*_*s*,*m*_|) than the rest, suggesting that they play more significant roles in making the predictions. Specifically, if the weight of an essential GO term for a particular subcellular location is larger than a certain positive threshold, it has higher confidence to indicate that the query protein resides in this subcellular location; on the contrary, if the weight is smaller than a certain negative threshold, it has higher confidence to indicate that the query protein does not belong to the corresponding subcellular location. We call these two kinds of GO terms as *significantly essential GO terms*.

### Circular networks for essential GO terms and subcellular locations

**Fig. 6:**
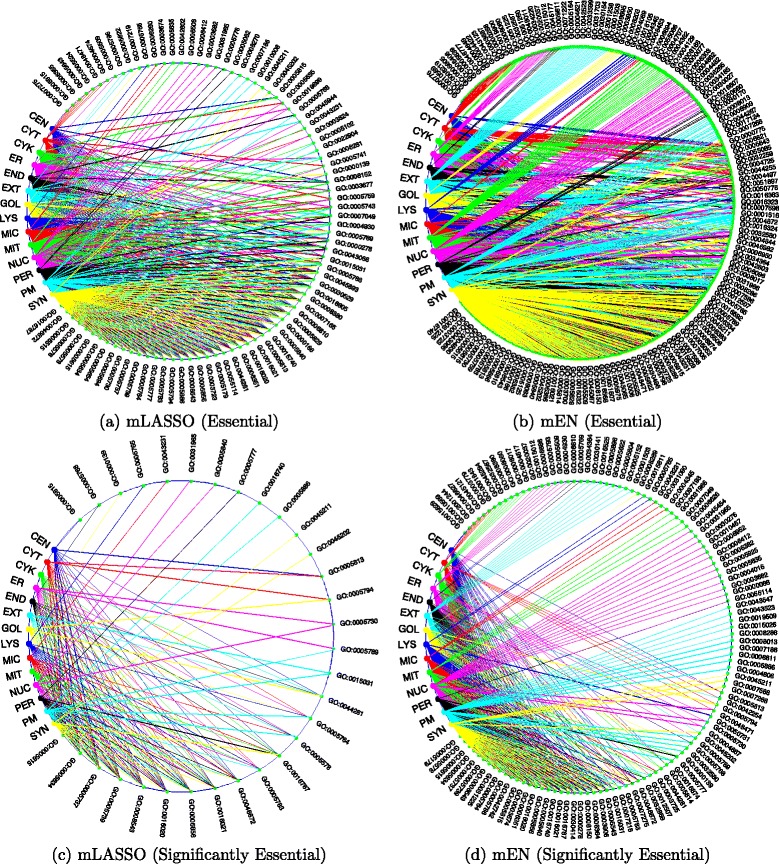
Networks showing the relationships between the essential GO terms and each subcellular location for (**a**) mLASSO and (**b**) mEN, and between the significantly essential GO terms and each subcellular location for (**c**) mLASSO and (**d**) mEN. In all figures, small green dots represent the GO terms and the large dots in different colors represent the 14 subcellular locations. A line connecting an essential GO term and a subcellular location denotes that the GO term contributes to the prediction of the subcellular location. On the contrary, if there is no line connecting an essential GO term with a particular subcellular location, then this GO term does not provide any information about the presence or absence of a protein in this particular subcellular location. *CEN*: centrosome; *CYT*: cytoplasm; *CYK*: cytoskeleton; *ER*: endoplasmic reticulum; *END*: endosome; *EXT*: extracellular; *GOL*: Golgi apparatus; *LYS*: lysosome; *MIC*: microsome; *MIT*: mitochondrion; *NUC*: nucleus; *PER*: peroxisome; *PM*: plasma membrane; *SYN*: synapse

To gain a comprehensive understanding of the relationships between the essential GO terms and the 14 subcellular locations for mLASSO and mEN, Fig. 6 shows the circular networks linking the essential GO terms and subcellular locations for four cases: (a) essential GO terms for mLASSO; (b) essential GO terms for mEN; (c) significantly essential GO terms for mLASSO; and (d) significantly essential GO terms for mEN. In all figures, small green dots represent the GO terms and the large dots in different colors represent the 14 subcellular locations. A line connecting an essential GO term and a subcellular location means that the GO term contributes to the prediction of the subcellular location. On the other hand, if there is no connection between an essential GO term and a subcellular location, then this GO term does not provide any information about the presence or absence of a protein in this particular subcellular location. Starting from the top-left green dot to the bottom-left green dot in clockwise direction, the degree of overlapping among the lines gradually increases, meaning that the number of subcellular locations to which a GO term contributes also gradually increases. For example, in Fig. 6(a), the first 7 GO terms (GO:0007275, GO:0006915, GO:0006355, GO:0005643, GO:0005524, GO:0048471 and GO:0004674) are indicative of *cytoplasm* only, i.e., suggesting whether a protein belongs to *cytoplasm* or not. Similarly, GO:0005509 can only indicates whether a protein is located in *endoplasmic reticulum* or not. On the other hand, GO:0005815 is indicative for both *centrosome* and *cytoskeleton*; GO:0005635 contributes to the prediction of both *cytoplasm* and *nucleus*. More aggressively, the last several GO terms, such as GO:0016787, GO:0046872 and GO:0005515, contribute to the prediction of all of the 14 subcellular locations. These essential GO terms are indicators of whether a protein resides in one or more subcellular location(s) or not. Similar conclusions can be drawn from Fig. 6(b). Compared to Fig. 6(a, b) has more essential GO terms to indicate the presence or absence of a query protein in the corresponding subcellular location. For readers’ convenience, all the essential GO terms found by mLASSO and mEN are listed in Section S3 and S4, respectively, of supplementary materials.

Figure 6(c) and (d) show the correlations between the significantly essential GO terms and the 14 subcellular locations (See the last section for the definition of *significantly essential GO terms*). There are 31 and 115 significantly essential GO terms shown in Fig. 6(c) and (d), respectively. As can be seen from Fig. 6(c), starting from the top-left to the bottom-left in clockwise direction, the first 12 GO terms are indicative of single-location only. The remaining 19 significantly essential GO terms are multi-location indicators. For readers’ convenience, all the essential GO terms found by mLASSO and mEN are listed in Sections S5 and S6, respectively, of supplementary materials.

Here we introduce the concept of the *key GO terms*, which are GO terms whose names are exactly the same as the names of subcellular locations according to the annotations. Interestingly, the *key GO terms* of some subcellular locations are both positive and negative indicators of multiple locations. For example, GO:0005813 is the key GO term for *centrosome*. A query protein with this term strongly indicates that it resides in *centrosome*, which is consistent with our results. However, mEN and mLASSO find that GO:0005813 is a negative indicator of *cytoplasm*, meaning that if a query protein is associated with this GO term, this protein is highly likely **not** to reside in *cytoplasm*. Actually, we have found (results not shown) that many significantly essential GO terms are not only positive indicators of some subcellular locations, but also negative indicators of other subcellular locations (indicating that proteins are unlikely to reside in particular subcellular locations). These GO terms allow us to find the proteins that cannot be co-located and the subcellular locations of these impossible combinations.

### Comparing with state-of-the-art predictors

Table 1 compares the performance of mLASSO and mEN against several state-of-the-art multi-label predictors on the human benchmark dataset. To the best of our knowledge, iLoc-Hum [68] is the best state-of-the-art predictor specializing for predicting multi-label human protein subcellular localization.^9^ mGOASVM [44] is not designed for predicting human protein subcellular localization, so we retrained it and applied the retrained mGOASVM to the human dataset. All of the predictors use some forms of GO vectors as features. From the classification perspective, iLoc-Hum use a multi-label KNN classifier; mGOASVM [44] uses a multi-label SVM classifier; and the proposed mLASSO and mEN use multi-label LASSO and EN classifiers, respectively.

**Table 1:** Comparing mLASSO and mEN with state-of-the-art multi-label predictors based on leave-one-out cross-validation on the human dataset

Label	Subcellular location	LOOCV Locative Accuracy (LA)			
		iLoc-Hum [68]	mGOASVM [44]	mLASSO	mEN
1	Centrosome	56/77 = 0.727	64/77 = 0.831	42/77 = 0.546	60/77 = 0.779
2	Cytoplasm	561/817 = 0.687	683/817 = 0.836	699/817 = 0.856	683/817 = 0.836
3	Cytoskeleton	27/79 = 0.342	44/79 = 0.557	29/79 = 0.367	32/79 = 0.405
4	Endoplasmic reticulum	166/229 = 0.725	193/229 = 0.843	194/229 = 0.847	190/229 = 0.830
5	Endosome	1/24 = 0.042	9/24 = 0.375	1/24 = 0.042	5/24 = 0.208
6	Extracellular	325/385 = 0.844	344/385 = 0.894	311/385 = 0.808	314/385 = 0.816
7	Golgi apparatus	99/161 = 0.615	131/161 = 0.814	118/161 = 0.733	128/161 = 0.795
8	Lysosome	56/77 = 0.727	71/77 = 0.922	62/77 = 0.805	74/77 = 0.961
9	Microsome	7/24 = 0.292	18/24 = 0.750	1/24 = 0.042	14/24 = 0.583
10	Mitochondrion	284/364 = 0.780	339/364 = 0.931	336/364 = 0.923	336/364 = 0.923
11	Nucleus	918/1021 = 0.899	931/1021 = 0.912	922/1021 = 0.903	923/1021 = 0.904
12	Peroxisome	20/47 = 0.426	43/47 = 0.915	34/47 = 0.723	39/47 = 0.830
13	Plasma membrane	277/354 = 0.783	288/354 = 0.814	267/354 = 0.754	266/354 = 0.751
14	Synapse	12/22 = 0.546	12/22 = 0.546	3/22 = 0.136	13/22 = 0.591
Overall Actual Accuracy (*OAA*)		2118/3106 = 0.682	2251/3106 = 0.725	2265/3106 = 0.729	2307/3106 = **0.743**
Overall Locative Accuracy (*OLA*)		2809/3681 = 0.763	3170/3681 = **0.861**	3019/3681 = 0.820	3077/3681 = 0.836
*Accuracy*		–	0.821	0.814	**0.827**
*Precision*		–	0.851	0.859	**0.869**
*Recall*		–	**0.888**	0.857	0.870
*F1*		–	0.853	0.843	**0.855**
*Micro F1*		–	0.835	0.826	**0.837**
*Macro F1*		–	0.740	0.638	**0.741**
*HL*		–	0.029	0.029	**0.028**

“–” means the corresponding references do not provide the related metrics. Note that *OAA* is the most stringent and objective among all the metrics. Data in bold represent the best result of the corresponding measures among all predictors

As shown in Table 1, mEN outperforms mLASSO in terms of all performance metrics, and it performs better than iLoc-Hum and mGOASVM in terms of *OAA, Accuracy, Precision, F1, Micro F1, Macro F1* and *HL*. Both mLASSO and mEN perform significantly better than iLoc-Hum. The *OAA* of mLASSO and mEN are 6 % (absolute) and 4 % higher than those of iLoc-Hum, respectively. When comparing with mGOASVM, the *OAA* of mEN is around 2 % (absolute) higher than that of mGOASVM, although a bit less than mGOASVM on the *OLA* and *Recall*. In terms of *Accuracy, Precision, F1, Micro F1, Macro F1* and *HL*, mEN performs better than mGOASVM. The results suggest that the proposed mEN performs better than the state-of-the-art classifiers. The individual locative accuracies of mEN are remarkably higher than that of iLoc-Hum, and are comparable to mGOASVM. The superiority of mEN over mLASSO is possibly caused by the fact that mEN selects more relevant GO terms (features) than mLASSO, and that the features selected by the former almost contains all of the information selected by the latter, as demonstrated in Fig. 3. This suggests that mLASSO is vulnerable to missing some important information when yielding parsimonious solutions.

## Predicting and interpreting novel proteins

To further exemplify how mEN predicts and interprets the subcellular localization of proteins, we collected several novel proteins as test proteins. These proteins, which include both single- and multi-location proteins, were experimentally determined and were added to Swiss-Prot between 19-Feb-2014 and 07-Jan-2015.^10^ The novelty of these proteins can impartially demonstrate the prediction powers of our proposed predictors. Table 2 shows the prediction results of the 7 novel proteins by mEN. As can be seen, although these proteins are totally new to our training dataset (created before 2009), all of them are correctly predicted, including one multi-location protein (E9PAV3). The essential GO terms that contribute to the prediction decisions are also shown in Table 2. A comparison between the essential GO terms in Fig. 6 and the last column in Table 2 reveals that not all of the essential GO terms contribute to the final predictions. For example, for the protein P0DMR3, only 8 out of 22 GO terms are useful for determining the subcellular localization. Interestingly, even if two proteins are predicted to the same subcellular location, the essential GO terms for the two proteins are not necessarily the same. For example, for D3DTV9 and C9JSJ3, although both of them are correctly predicted to locate in *nucleus*, their essential GO terms are completely different. And there is no significantly essential GO terms for the protein D3DTV9. This suggests that the predictions made by mEN do not always rely on significantly essential GO terms.

**Table 2:** Prediction results of 7 novel proteins by mEN

AC	Date of creation	Ground-truth location(s)	Prediction results	GO total number	Essential GO terms
D3DTV9	26-Nov-2014	Nucleus	Nucleus	13	GO:0000166, GO:0016787, GO:0003676, GO:0003723, GO:0004386, GO:0046872, GO:0051607, GO:0005524
E9PAV3	19-Feb-2014	Cytoplasm, Nucleus	Cytoplasm, Nucleus	9	GO:0015031, GO:0003677, GO:0005634, GO:0005737, GO:0006351, GO:0006355, GO:0006810
B7ZW38	26-Nov-2014	Nucleus	Nucleus	5	GO:0000166, GO:0030529, GO:0003676, GO:0003723, GO:0005634
P0DMR3	07-Jan-2015	Cytoplasm	Cytoplasm	22	GO:0000166, GO:0016740, GO:0016874, GO:0003824, GO:0046872, GO:0005524, GO:0005575, GO:0008152
P0DML3	09-Jul-2014	Extracellular	Extracellular	6	GO:0046872, GO:0005179, GO:0005576, GO:0007165
P0DMN0	03-Sep-2014	Cytoplasm	Cytoplasm	16	GO:0016740, GO:0030968, GO:0044267, GO:0044281, GO:0005737, GO:0005829, GO:0006629, GO:0006805
C9JSJ3	29-Oct-2014	Nucleus	Nucleus	4	GO:0003677, GO:0005634, GO:0006351, GO:0006355

*AC*: UniProtKB accession number; *Ground-truth location(s)*: the experimentally-validated actual subcellular location(s); *GO Total Number*: the total number of GO terms retrieved for a given query protein

**Fig. 7:**
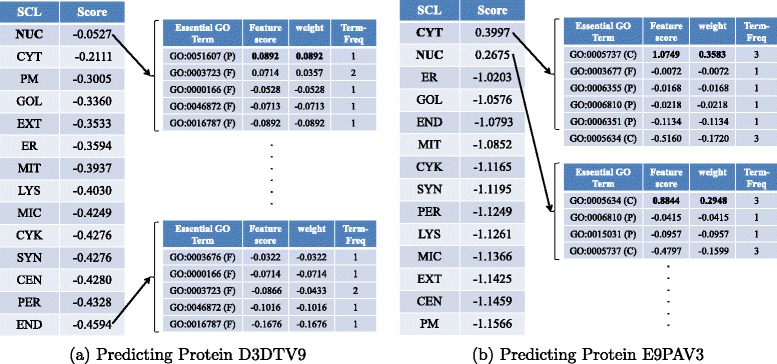
Examples showing how mEN interprets subcellular localization of (**a**) a single-location protein (D3DTV9) and (**b**) a multi-location protein (E9PAV3). *SCL*: subcellular location; *Score*: the score determined in Eq. 14; *Feature Score*: the score that each essential GO term contributes to the final prediction; *Term-freq*: the frequency of occurrence of an essential GO term; *C*: cellular component; *F*: molecular function; *P*: biological process; *CEN*: centrosome; *CYT*: cytoplasm; *CYK*: cytoskeleton; *ER*: endoplasmic reticulum; *END*: endosome; *EXT*: extracellular; *GOL*: Golgi apparatus; *LYS*: lysosome; *MIC*: microsome; *MIT*: mitochondrion; *NUC*: nucleus; *PER*: peroxisome; *PM*: plasma membrane; *SYN*: synapse

Figure 7 demonstrates how researchers can use mEN to interpret the prediction results of query proteins. Figure 7(a) shows the scores produced by Eq. 14 in descending order using the query protein D3DTV9 (Table 2) as input, where (P) and (F) stand for biological process and molecular function categories, respectively. Also, the columns “Weight” and “Term-Freq” represent non-zero elements of $\tilde {\boldsymbol {\beta }}^{\text {en}}_{m}$ in Eq. 13 and $\textbf {x}^{s}_{t}$ in Eq. 11, and the column “Feature Score” represents the product of Weight and Term-Freq. The higher the feature score, the more contribution is the corresponding GO term to the prediction result. Since all of the 14 scores are negative, the number of subcellular locations is predicted to be 1 and the subcellular location is determined by the maximum score, which corresponds to *nucleus*. The scores and weights for the essential GO terms in *nucleus* and *endosome* are also shown in the right panel of Fig. 7(a).^11^ As can be seen, only 5 out of 13 (See Table 2) essential GO terms contribute to the scores corresponding to *nucleus*. More interestingly, the top essential GO term (GO:0051607) belongs to biological process (P), while the remaining 4 belong to molecular function (F) and none of them belongs to the cellular-component category. This suggests that GO terms from the categories of molecular function and biological process can also play key roles in determining the subcellular localization of proteins. Figure 7(b) shows the case for a multi-location protein (E9PAV3). Evidently, there are two positive scores, respectively determined by 6 and 4 essential GO terms. Thus, E9PAV3 is predicted to co-locate in *cytoplasm* and *nucleus*. This demonstrates that mEN can predict multi-location proteins. Moreover, the sets of essential GO terms to determine the presence of E9PAV3 in *cytoplasm* and *nucleus* are different.

## Discussion

### Mapping non-essential GO terms to essential GO terms

Some researchers are concerned that not all proteins (especially those novel proteins) are associated with essential GO terms selected by mLASSO (or mEN). In this case, the feature vectors for these proteins will be null, which is detrimental for prediction performance. To address this problem, we have proposed a hierarchical-information based approach to map the non-essential GO terms to essential GO terms. Because the GO terms in each taxonomy (cellular components, molecular functions or biological processes) are hierarchically organized within a directed acyclic graph (DAG), GO terms in the same taxonomy are not independent with each other. In this case, non-essential GO terms are hierarchically correlated with essential GO terms within the same taxonomy. Therefore, it is feasible and also conducive to map the non-essential GO terms to the essential GO terms based on their hierarchical structural relationships.

We adopt our recently proposed GO mapping method to perform the mapping. Here, we briefly outline the procedure. For implementation details, readers may refer to Eqs. 5 and 6 of [69]. Denote *G* as a GO term (either non-essential or essential) and *E* as an essential GO term. Assume that *G* is one of the GO terms associated with a protein and that the number of occurrences of *G* for that protein is *f*_*G*_. Then, mapping *G* to *E* is equivalent to finding the contribution of *G* to the *effective* number of occurrences of *E*, where the contribution is computed from the depth distance between *G* and *E* as shown in Algorithm 1. The effective number of occurrences of *E* is the sum of the contribution of all GO terms associated with the query protein. This procedure is repeated for every essential GO terms to form a vector comprising the effective numbers of occurrences of all essential GO terms. This GO mapping method effectively solves the null-vector problem, because as long as one of the GO term associated with the query protein is close enough to any essential GO term in the GO DAG, the resulting vector will not be null.



Table 3 investigates the impacts of the hierarchical-information based (HIB) technique on mLASSO and mEN based on LOOCV on the human dataset. As can be seen, the HIB technique can improve the performance of mLASSO in terms of all performance metrics. On the contrary, mEN with HIB performs slightly worse than mEN without HIB. This is understandable because compared to mEN, mLASSO selects fewer GO terms so that information in some of the discarded GO terms is lost. The HIB method can partially retain this lost information in the HIB vectors (Eq. 5 of [69]) through the structural relationship between the discarded terms and the selected terms, leading to improved performance. On the other hand, because mEN selects more essential GO terms and thus less information is lost, the HIB technique is less helpful to mEN. The performance of mEN with the HIB technique is even slightly worse than that of mEN without HIB due to different representations of the feature information. More interestingly, except for *Macro F1*, the performance of mLASSO with HIB outweighs that of mEN in all performance metrics.

**Table 3:** Impacts of the hierarchical-information based (HIB) technique on mLASSO and mEN based on leave-one-out cross-validation (LOOCV) on the human dataset

Measures	mLASSO		mEN	
	without HIB	with HIB	without HIB	with HIB
*OAA*	0.729	**0.748**	0.743	0.742
*OLA*	0.820	**0.846**	0.836	0.825
*Accuracy*	0.814	**0.833**	0.827	0.821
*Precision*	0.859	**0.874**	0.869	0.866
*Recall*	0.857	**0.879**	0.870	0.860
*F1*	0.843	**0.862**	0.855	0.849
*Micro F1*	0.826	**0.844**	0.837	0.831
*Macro F1*	0.638	0.676	**0.741**	0.667
*HL*	0.029	**0.027**	0.028	0.029

Note that *OAA* is the most stringent and objective among all the metrics. Data in bold represent the best result of the corresponding measures among all predictors

### Categorical significance of GO terms on prediction

To investigate the contributions of GO terms from different categories to the prediction performance of mLASSO and mEN, we have compared the performance of using GO terms for the following cases: (1) *All*: all GO terms are used; (2) *CC + MF*: GO terms from cellular components (CC) and molecular functions (MF) are used; (3) *CC + BP*: GO terms from CC and biological processes (BP); and (4) *MF + BP*: GO terms from MF and BP are used. The results are shown in Table 4. As can be seen, for both mLASSO and mEN, Case (1) performs the best among all the four cases, due to using GO terms from all of the three categories. The results indicate that GO terms from all of the three categories are conducive to the predictions. Moreover, the performance of Cases (2) and (3) remarkably surpass that of Case (4), which suggests that CC GO terms are more far more significant than GO terms from the other two categories for the predictions. Besides, although Case (4) performs the worst among the four cases, it also demonstrates that GO terms from MF and BP are also useful for the predictions.

**Table 4:** Significance of GO terms from different categories on the performance of mLASSO and mEN based on leave-one-out cross-validation (LOOCV) on the human dataset

Measures	mLASSO				mEN			
	All	CC + MF	CC + BP	MF + BP	All	CC + MF	CC + BP	MF + BP
*OAA*	0.729	0.662	0.654	0.385	**0.743**	0.621	0.640	0.440
*OLA*	0.820	0.726	0.715	0.436	**0.836**	0.686	0.701	0.492
*Accuracy*	0.814	0.733	0.724	0.446	**0.827**	0.690	0.709	0.506
*Precision*	0.859	0.782	0.773	0.500	**0.869**	0.739	0.760	0.560
*Recall*	0.857	0.759	0.747	0.457	**0.870**	0.712	0.730	0.521
*F1*	0.843	0.758	0.748	0.469	**0.855**	0.713	0.733	0.528
*Micro F1*	0.826	0.750	0.741	0.462	**0.837**	0.711	0.728	0.516
*Macro F1*	0.638	0.435	0.426	0.212	**0.741**	0.410	0.427	0.346
*HL*	0.029	0.041	0.042	0.086	**0.028**	0.047	0.044	0.078

Note that *OAA* is the most stringent and objective among all the metrics. *CC*: cellular components; *MF*: molecular functions; *BP*: biological processes. Data in bold represent the best result of the corresponding measures among all predictors

## Conclusions

In this paper, we proposed and compared two sparse multi-label predictors, namely mLASSO and mEN, which can predict as well as interpret the subcellular localization(s) of both single- and multi-location proteins. Given a query protein, its feature vector is constructed by exploiting the GO frequency information in the ProSeq-GO database. By using the one-vs-rest LASSO and EN classifiers, 87 and 429 out of 8,000+ GO terms are selected, respectively. Based on these selected essential GO terms, the interpretability is analyzed for both algorithms.

This paper has the following key contributions: (1) Both mEN and mLASSO are interpretable and perform remarkably better than existing state-of-the-art predictors; (2) mEN selects more relevant GO terms than mLASSO, and meanwhile outperforms mLASSO; (3) Experimental results for both methods are consistent with biological annotations, i.e., the key GO terms play greater roles in determining subcellular localization of proteins; (4) Like cellular-component GO terms, GO terms from the categories of molecular functions and biological processes also contribute to the prediction; (5) Essential GO terms can be either single-location contributive or multi-location contributive to the prediction, and the contributions can be positive on a subcellular location while be negative on other subcellular locations. For readers’ convenience, the SpaPredictor web-server and the supplementary materials of this paper are available online at http://bioinfo.eie.polyu.edu.hk/SpaPredictorServer/.

## Methods

### Feature extraction

#### 1) Creation of compact databases

The applicability of existing GO-based approaches is limited by the availability of GO information for query proteins, especially for novel proteins. Conventionally, given a query protein, if its accession number (AC) cannot be associated with any GO term in the GOA database, BLAST [53] was used to retrieve its top homologous protein which is supposed to be annotated in the GOA database, and thus whose AC can be associated with a set of GO terms. In this case, the homologous GO information can be transferred to the query protein. However, this strategy will become ineffective when no GO information can be retrieved from the top homolog. In such case, some predictors use back-up methods that rely on other features, such as pseudo-amino-acid composition [14] and sorting signals [70]; some predictors [34, 44] use a successive-search strategy to avoid null GO vectors. Nonetheless, these strategies may lead to poor performance and increase computation and storage complexity.

To address this problem, similar to our earlier work [46, 71], we created two small yet efficient databases: ProSeq and ProSeq-GO. The former is a sequence database extracted from the Swiss-Prot database and the latter is a GO-term database extracted from the GOA database. Detailed descriptions of the procedures can be found in [46]. By using ProSeq and ProSeq-GO, we can not only guarantee that every query protein can associate with at least one GO term, but also significantly reduce the memory consumption.

#### 2) Construction of GO vectors

The construction of feature vectors involves two steps: (1) retrieval of GO terms; and (2) construction of GO vectors.

For the retrieval of GO terms, given a query protein, its amino acid sequence is presented to BLAST [53] to find its homologs in the ProSeq database. The homologous ACs are then used as keys to search against the ProSeq-GO database.

For the construction of GO vectors, given a dataset, the GO terms of all of its proteins are retrieved by the procedures described above. Because term-frequency (TF) based GO vectors [34, 44] are found to perform better than the conventional 1-0 vectors, we adopted the TF method to construct GO vectors. Specifically, suppose the number of distinct GO terms for the dataset of interest is *T*, then the GO vector **q**_*i*_ of the *i*-th protein  is defined as:

(5) $$ \textbf{q}_{i}=[f_{i,1},\ldots,f_{i,j},\ldots,f_{i,T}]^{\textsf{T}},   $$

where *f*_*i*,*j*_ is the number of occurrences of the *j*-th GO term (term-frequency) in the *i*-th protein sequence. Detailed information about GO vectors can be found in [34, 44].

### Multi-label sparse-regression based classifiers

An interesting and useful property of sparse regression models is that they can produce “parsimonious” solutions that enable us to find a set of features that are the most relevant to the problem (target variables) being addressed. Usually, sparse regressions are achieved by imposing regularized constraints on the features. Two common linear sparse regression models are LASSO [72] and elastic net (EN) [67]. The former is short for Least Absolute Shrinkage and Selection Operator, which is an *L*_1_-regularized linear regression model. The *L*_1_ constraint forces the weights of some features to exactly zero [73], and hence LASSO can automatically select relevant features. The latter is an (*L*_1_+*L*_2_)-regularized linear regression model. The convex combination of *L*_1_ and *L*_2_ penalties can yield sparse representations similar to LASSO, while encouraging correlated features to be selected or deselected together [67]. LASSO can be regarded as a special case of EN, which is explained in the following section.

LASSO has been applied to many bioinformatics domains, such as gene regulation network analysis [73], microRNA-target regulatory network construction [74], inflammation-cancer relationship analysis [75] and plant gene detection [76]. EN has been extensively used in various aspects of computational biology, such as genetic trait prediction [77], ICU mortality risk detection [78], single nucleotide polymorphism (SNP) selection [79], etc.

#### 1) Objective functions of sparse regressions

Sparse regressions are applicable to classification. Suppose for a two-class single-label problem, we are given a set of training data $\{\textbf {x}_{i},y_{i}\}_{i=1}^{N}$, where $\textbf {x}_{i}\in {\mathcal {R}}^{T}$ and *y*_*i*_∈{−1,1}. In our case, **x**_*i*_=**q**_*i*_, where **q**_*i*_ is defined in Eq. 5.

Generally speaking, a LASSO model is to impose an *L*_1_ regularization to ordinary least squares (OLS):

(6) $$ {}l^{\text{lasso}}(\boldsymbol{\beta}) = \sum_{i=1}^{N}(y_{i}-f(\textbf{x}_{i}))^{2}=\sum_{i=1}^{N}\left(y_{i}- \varepsilon_{0}-\sum_{j=1}^{T}\beta_{j}x_{i,j}\right)^{2},  $$

subject to

$$\sum_{j=1}^{T} |\beta_{j}| \leq t, $$

where ***β***=[*β*_1_,…,*β*_*j*_,…,*β*_*T*_]^T^ is the LASSO vector to be optimized, *t*>0 is a parameter controlling the shrinkage level applied to ***β***, *ε*_0_ is a bias,^12^ and *x*_*i*,*j*_ is the *j*-th element of **x**_*i*_. Equation 6 is equivalent to minimizing the following equation:

(7) $$ l^{\text{lasso}}(\boldsymbol{\beta}) = \sum_{i=1}^{N} \left(y_{i}-\boldsymbol{\beta}^{\textsf{T}}\textbf{x}_{i}\right)^{2} + \lambda \sum_{j=1}^{T}|\beta_{j}|,  $$

where *λ*>0 is a penalized parameter controlling the degree of regularization. Equation 7 is a convex optimization problem, and can be efficiently solved. We adopted the least angle regression (LARS) method to solve this problem. Detailed descriptions of the LARS algorithm can be found in [80].

EN is to impose an (*L*_1_+*L*_2_)-style regularization on Eq. 6. Thus, the object function of EN is:

(8) $$ l^{\text{en}}(\boldsymbol{\beta}) = \sum_{i=1}^{N} \left(y_{i}-\boldsymbol{\beta}^{\textsf{T}}\textbf{x}_{i}\right)^{2} + \lambda \sum_{j=1}^{T}|\beta_{j}| + \gamma \sum_{j=1}^{T} {\beta_{j}}^{2},  $$

where *λ*>0 and *γ*>0 are the penalty parameters controlling the ridge regression penalty and lasso penalty, respectively. As can be seen, when *λ*=0, Eq. 8 becomes simple ridge regression; when *γ*=0, Eq. 8 is exactly the same as Eq. 7. Besides, by simple transformation, Eq. 8 can be converted to an equivalent LASSO-style problem on augmented data [67]. Because of this property, Eq. 8 can be solved by the same way as LASSO by absorbing the *L*_2_-norm term into the objective function. Detailed descriptions of the solutions can be found in [67]. In this work, the LASSO and the elastic net algorithms were implemented by using the functions *lasso.m* and *elasticnet.m*, respectively, in the SpaSM package [81]. This package can be downloaded from http://www2.imm.dtu.dk/projects/spasm/.

#### 2) Multi-label LASSO/EN for feature selection

In an *M*-class multi-label problem, the training data set is written as $\{\textbf {x}_{i},{\mathcal {Y}}_{i}\}_{i=1}^{N}$, where $\textbf {x}_{i}\in {\mathcal {R}}^{T}$ and ${\mathcal {Y}}_{i} \subset \{1,2,\ldots,M\}$ is a set which may contain one or more labels.

For the multi-label LASSO (EN), *M* independent binary one-vs-rest LASSOs (ENs) are trained, one for each class. The labels $\{{\mathcal {Y}}_{i}\}_{i=1}^{N}$ are converted to *transformed labels* [44] *y*_*i*,*m*_∈{−1,1}, where *i*=1,…,*N*, and *m*=1,…,*M*. Then, the LASSO and EN estimate vectors for the *m*-th class is given by:

(9) $$ {}\hat{\boldsymbol{\beta}}^{\text{lasso}}_{m} = \mathop{arg min}_{\boldsymbol{\beta}_{m}} \left \{ \sum_{i=1}^{N} \left(y_{i,m}-\boldsymbol{\beta}_{m}^{\textsf{T}}\textbf{x}_{i}\right)^{2} + \lambda_{m} \sum_{j=1}^{T}|\beta_{j,m}| \right \},  $$

and

(10) $$ {\footnotesize{} \begin{aligned} \hat{\boldsymbol{\beta}}^{\text{en}}_{m} = \mathop{arg min}_{\boldsymbol{\beta}_{m}} \left \{ \sum_{i=1}^{N} \left(y_{i,m}-\boldsymbol{\beta}_{m}^{\textsf{T}}\textbf{x}_{i}\right)^{2} + \lambda_{m} \sum_{j=1}^{T}|\beta_{j,m}| + \gamma_{m} \sum_{j=1}^{T}{\beta_{j,m}}^{2} \right \}, \end{aligned}}  $$

respectively. In Eq. 9, *m*=1,…,*M*, $\{y_{i,m}\}_{i=1}^{N} \in \{-1,1\}$, and *λ*_*m*_ is the penalized parameter for the *m*-th class. And in Eq. 10, *m*=1,…,*M*, $\{y_{i,m}\}_{i=1}^{N} \in \{-1,1\}$, *λ*_*m*_ and *γ*_*m*_ are the *L*_1_ penalized parameter and the *L*_2_ penalized parameter for the *m*-th class, respectively. Since *L*_1_ regularization tends to force some weights $\{\beta _{j,m}\}_{j=1}^{T}$ for the *m*-th class to exactly zero, both LASSO and EN can be used for feature selection. The difference between LASSO and EN is the degree of parsimoniousness in the solution.

The GO vectors obtained from Eq. 5 are used for training multi-label one-vs-rest LASSO classifiers. For an *M*-class problem (here *M* is the number of subcellular locations), *M* independent binary LASSO classifiers are trained, one for each class. After training, the union of those GO terms whose weights are not zero in any one of the *M* classes constitutes the selected features. LASSO can impressively remove those irrelevant features (or GO terms). Suppose *S* out of the *T* weights are nonzero. They are defined as {*β*_*s*,*m*_}_*s*={1,…,*S*},*m*={1,…,*M*}_ and their corresponding GO terms are called *essential GO terms*. In fact, in our experiments, through the proposed multi-label LASSO classifiers, 87 out of 8110 GO terms were selected. This means that only around 1 % of the GO terms are *essential GO terms* and the weights for about 99 % of the 8110 GO terms are exactly zero.

Similar procedures were applied to multi-label EN. Through the proposed multi-label EN classifiers, 429 out of 8110 GO terms were selected. This means that around 5 % of the GO terms are *essential GO terms* and the weights for about 95 % of the 8110 GO terms are exactly zero.

#### 3) Multi-label LASSO/EN for classification

Besides feature selection, LASSO and EN can also be used for classification. Specifically, given the *t*-th query protein  the feature vector $\textbf {x}_{t} \in {\mathcal {R}}^{T}$ defined in Eq. 5 is obtained. Then, the elements of **x**_*t*_ with non-zero weights *β*_*j*,*m*_ (in Eq. 9 for LASSO and in Eq. 10 for EN) are selected to form a low-dimensional feature vector represented by $\textbf {x}^{s}_{t} \in {\mathcal {R}}^{S}$, where *S*<*T* is the number of essential GO terms. Similarly, for an *M*-class problem, *M* independent binary LASSO (EN) classifiers are trained, one for each class. Then, the score of the *m*-th LASSO (EN) is: 
(11)

where ${\tilde {\boldsymbol {\beta }}_{m}}$ for LASSO and EN are given by

(12) $$ {}\tilde{\boldsymbol{\beta}}^{\text{lasso}}_{m} = \mathop{arg min}_{\boldsymbol{\alpha}_{m}} \left \{ \sum_{i=1}^{N} \left(y_{i,m}-\boldsymbol{\alpha}_{m}^{\textsf{T}}\textbf{x}^{s}_{i}\right)^{2} + \lambda_{m} \sum_{j=1}^{S}|\alpha_{j,m}| \right \},  $$

and

(13) $$ {\footnotesize{} \begin{aligned} \tilde{\boldsymbol{\beta}}^{\text{en}}_{m} = \mathop{arg min}_{\boldsymbol{\alpha}_{m}} \left \{ \sum_{i=1}^{N} \left(y_{i,m}-\boldsymbol{\alpha}_{m}^{\textsf{T}}\textbf{x}^{s}_{i}\right)^{2} + \lambda_{m} \sum_{j=1}^{S}|\alpha_{j,m}| + \gamma_{m} \sum_{j=1}^{S}{\alpha_{j,m}}^{2} \right \}, \end{aligned}}  $$

respectively, where ***α***_*m*_=[*α*_1,*m*_,…,*α*_*j*,*m*_,…,*α*_*S*,*m*_]^T^ is the weight vector to be optimized and $\textbf {x}^{s}_{i} \in {\mathcal {R}}^{S}$ is the feature vector for the *i*-th training protein. Note that ${\tilde {\boldsymbol {\beta }}_{m}}$ in both equations are obtained based only on the training data.

To predict the subcellular locations of datasets containing both single-label and multi-label proteins, a decision scheme for multi-label LASSO (EN) classifiers should be used. Unlike the single-label problem where each protein has one predicted label only, a multi-label protein should have more than one predicted labels. In this paper, we used the decision scheme described in mGOASVM [44]. In this scheme, the predicted subcellular location(s) of the *i*-th query protein are given by: 
(14)

For ease of presentation, we refer to the two proposed predictors as mLASSO and mEN, respectively.

## Endnotes

^1^http://www.proteinatlas.org/

^2^http://www.geneontology.org

^3^http://prosite.expasy.org/

^4^http://www.ebi.ac.uk/GOA

^5^http://www.uniprot.org/

^6^ Locative proteins are defined as follows. If a protein exists in two different subcellular locations, it will be counted as two locative proteins; if a protein coexists in three locations, then it will be counted as three locative proteins; and so forth.

^7^ In our case, *N*=3106 for the human dataset.

^8^ Specific weights $\{\beta _{s,m}\}_{s \in {\mathcal S}, m=\{1,\ldots,M\}}$ of each subcellular location for mLASSO and mEN can be found in Section S1 and S2, respectively, of the supplementary materials.

^9^ Hum-mPLoc 2.0 [43] performs worse than iLoc-Hum, and only the *OLA* is provided in [43]. Therefore, we do not report the performance of Hum-mPLoc 2.0 here.

^10^ Note that because the number of novel reviewed human proteins that were added to Swiss-Prot after 2014 is too small to constitute a meaningful test set, we used some representative novel proteins to test mEN instead.

^11^ The scores and weights for the essential GO terms for all of the 14 subcellular locations can be seen by inputing the query protein sequence to our SpaPredictor web-server.

^12^ For ease of presentation, we omitted the bias in equations thereafter.
